# Activation of Estrogen Receptor-α by E2 or EGF Induces Temporally Distinct Patterns of Large-Scale Chromatin Modification and mRNA Transcription

**DOI:** 10.1371/journal.pone.0002286

**Published:** 2008-05-28

**Authors:** Valeria Berno, Larbi Amazit, Cruz Hinojos, Jeannie Zhong, Maureen G. Mancini, Zelton Dave Sharp, Michael A. Mancini

**Affiliations:** 1 Molecular and Cellular Biology, Baylor College of Medicine, Houston, Texas, United States of America; 2 Molecular Medicine, Institute of Biotechnology, University of Texas Health Science Center San Antonio, San Antonio, Texas, United States of America; University of Hong Kong, China

## Abstract

Estrogen receptor-α (ER) transcription function is regulated in a ligand-dependent (e.g., estradiol, E2) or ligand-independent (e.g., growth factors) manner. Our laboratory seeks to understand these two modes of action. Using a cell line that contains a visible prolactin enhancer/promoter array (PRL-HeLa) regulated by ER, we analyzed ER response to E2 and EGF by quantifying image-based results. Data show differential recruitment of GFP-ER to the array, with the AF1 domain playing a vital role in EGF-mediated responsiveness. Temporal analyses of large-scale chromatin dynamics, and accumulation of array-localized reporter mRNA over 24 hours showed that the EGF response consists of a single pulse of reporter mRNA accumulation concomitant with transient increase in array decondensation. Estradiol induced a novel cyclical pattern of mRNA accumulation with a sustained increase in array decondensation. Collectively, our work shows that there is a stimuli-specific pattern of large-scale chromatin modification and transcript levels by ER.

## Introduction

The estrogen receptor-α (ER) integrates signals from different stimuli in its role as a transcription regulator. The transcriptional activity of ER is regulated directly through binding of estrogenic or anti-estrogenic molecules [1]. Indirectly it is regulated by growth factor signaling pathways [2], [3]. Although there is overlap with direct activation, growth factors activate kinase cascades resulting in phosphorylation and activation of the ER, which is distinguishable from ligand activation [4]. Indeed, cross talk between the regulatory pathways indicates that direct and indirect regulation of ER are not mutually exclusive [4]–[6]. Epidermal growth factor (EGF) and its receptor, EGFR, initiate an important signaling cascade leading to an activated ER [7]. It has been hypothesized that the combined over-stimulation of ER and the EGF receptor (EGFR) may provide a strong stimulus for breast tumor growth and may contribute to the resistance of tumor cells to antagonist therapy. The molecular mechanism of this activation remains poorly understood and is an area of continued study [8]–[10].

EGF signaling is initiated by binding and activation of the EGFR at the plasma membrane [11]. Tyrosine autophosphorylation by EGFR initiates multiple kinase cascades, targets of which include the ER. EGF induces ER-dependent stimulation of estrogen responsive element (ERE) reporter expression [12]. In this model, the extracellular EGF signal is transduced to genes regulated by the ER, the physiological relevance of which is underlined by estrogen-like effects of EGF on the mouse uterus do not occur in ER-deficient transgenic mice [13].

ERK1 and ERK2, kinases belonging to the MAPK pathway, have been shown to phosphorylate the ER at serine 118 in the activation function-1 (AF-1) domain of ER [3], [14]. This post-translational modification has a strong impact on ER-mediated transcriptional activation induced by both direct (estradiol) and indirect signaling [15], [16]. Interestingly, ER phosphorylation at serine 118 is also a marker of an activated ER signaling pathway in breast cancer, and provides a precise biomarker of responsiveness to endocrine therapy [17], [18]. Therefore, elucidation of the mechanism of EGF-dependant activation of ER could be important in the development of new therapeutic targets for overcoming the resistance of breast tumor cells to hormone-therapy.

We have developed a model system, PRL-HeLa, for the single-cell study of multiple mechanistic aspects of ER regulation of transcription [19]. This cell line contains a multi-copy integrated prolactin (PRL) enhancer/promoter reporter construct, which is responsive to E2. When ER is expressed as a GFP-fusion protein (GFP-ER), the integration site can be easily visualized allowing spatial and temporal analyses of promoter/enhancer targeting by ER, large-scale chromatin modification and accumulation of reporter mRNA. In our initial studies, we used PRL-HeLa to examine ligand-dependent ER regulation [19]. Treatment of these cells with E2 induces an ER-dependent large-scale chromatin decondensation, coactivator recruitment and maximal reporter mRNA accumulation. Conversely, treatment with the anti-estrogen 4-hydroxy-tamoxifen (4HT) induces large-scale chromatin condensation, abrogates coactivator recruitment, concomitant with a marked repression of reporter gene transcription. PRL-HeLa can be used to simultaneously examine several mechanistic aspects of ER transcription regulation at early (minutes) or late (hours) stages.

ER is an important regulator of pituitary function, and the expression of the prolactin gene is also responsive to other factors, including EGF [20]. Accordingly, we sought to compare indirect (E2)- and indirect (EGF)-responsive regulation of ER-mediated transcription using our PRL-HeLa model system. Using quantitative automated imaging [21], our studies reveal differential recruitment of GFP-ER to the PRL array, sustained, maximum chromatin decondensation over 24 hours in E2 treated cells, accompanied by cyclic levels of reporter mRNA accumulation at the PRL-array. In contrast, EGF treatment induces a single pulse of ER-dependent chromatin decondensation and mRNA accumulation. These studies indicate a previously unknown difference between ligand-dependent and -independent control of chromatin decondensation by ER, coincident with different transcriptional responses.

## Results

### EGF-dependent ER promoter targeting

**Figure 1:**
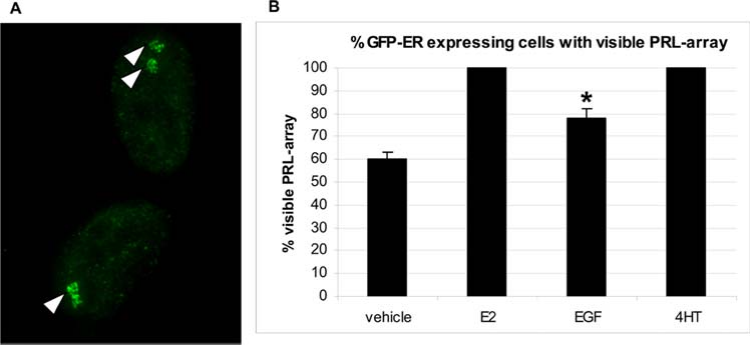
Estrogen receptor promoter binding in PRL-HeLa cells. GFP-ER was transiently expressed in PRL-HeLa cells and then treated with ethanolic vehicle, 17-β estradiol, EGF or 4-hydroxytamoxifen for 2 hours prior to fixation. Each ligand concentration was 10 nM except for EGF, which was 100 ng/ml. A. Representative images of PRL-HeLa cells treated with EGF. Arrowheads point to visible arrays in two cells. One of the cells has two fluorescent foci/arrays, while the other has one larger focus. B The percentage of each cell population that has visible accumulation at the array in response to each treatment has been calculated and graphed (n>200). Bars indicate Standard Deviations from 3 different experiments. Student t-test was performed for each bar compared to vehicle treatment. *p<0.05.

PRL-HeLa cells transiently transfected with a GFP-ER expression vector were maintained for 48 hours in a hormone-free medium, and were then treated with 10 nM E2, 100 ng/ml EGF or 10 nM 4HT. PRL-arrays were visualized as bright foci of nuclear fluorescence in GFP-ER-expressing cells (**Figure 1A**, **arrows**), These foci co-localize with the integrated PRL reporter chromatin by DNA and RNA fluorescence *in situ* hybridization (FISH) [19]. Transient transfection results in a range of protein expression [19], [21], and analyses of individual cells require careful selection based upon fluorescence (GFP-ER) level [21]. To avoid overexpression issues, array responses were assayed in cells expressing GFP-ER at levels of <1.5 fold compared to endogenous ER in MCF-7 cells [19]. Relative to vehicle control, both E2 and 4HT treatments result in an increase in the percentage of cells with visible PRL arrays in GFP-ER expressing cells (60% versus 100% and 100%, respectively, **Figure 1B**). EGF treatment (indirect activation) resulted in 78% of GFP-expressing cells demonstrating visible arrays, which is statistically greater than vehicle control, but lower in percentage than E2-treated (direct activation).

**Figure 2:**
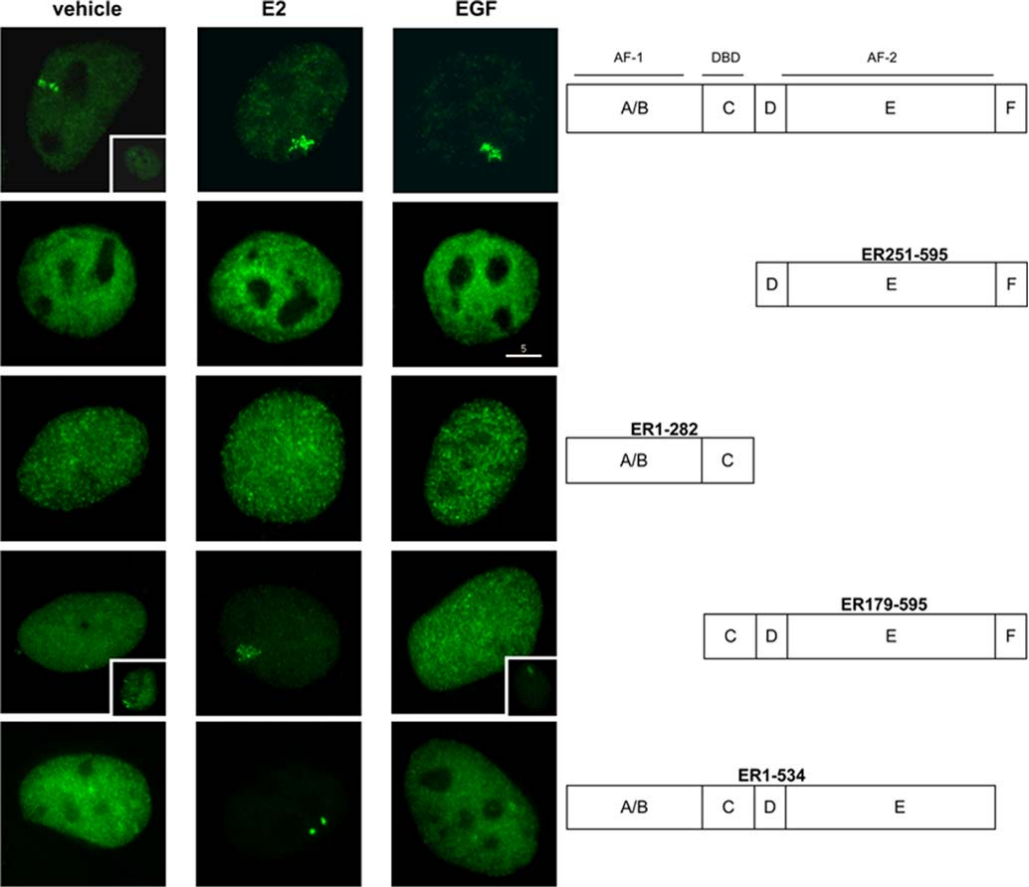
Role of AF-1 and AF-2 ER domains in promoter binding. Representative images of PRL-HeLa cells transiently expressing the indicated deletion mutants and treated with either ethanolic vehicle, E2 or EGF for 2 hours (see Table 1). The inset images are representative of fluorescence images observed in a minority of cells. The size bar is in microns. All images were captured so as to optimize the intensity of the GFP-ER.

To determine the functional domains of ER required for EGF-mediated array responses, we expressed previously described deletion mutants (**Figure 2** and [19]). Consistent with previous observations [19], expression of mutated ER in which the AF1 and DNA binding domain (GFP-ER_251–595_), or the ligand-binding domain (GFP-ER_1–282_) are deleted resulted in diffuse nuclear fluorescence (no visible foci) regardless of treatment (**Figure 2** and **Table 1**). Expression of receptor in which the AF-1 domain (GFP-ER_179–595_) is removed demonstrated PRL-array targeting in the presence of E2, but not EGF (**Figure 2**). Therefore, the activity of the AF1 transactivation domain in the ER is necessary, but not sufficient, for optimal EGF-induced association with the PRL-array.

**Table 1:** Promoter targeting and chromatin status in PRL-HeLa for ER deletion mutants.

	wt	251–595	1–282	179–595	1–534
**vehicle**	60% (±2.8) decondensed	0%	0%	22.5% (±3.5)* decondensed	0%
**E2**	100% decondensed	0%	0%	89.5% (±0.7)*,† decondensed	100% condensed
**EGF**	78% (±4) decondensed	0%	0%	19% (±2.8)* decondensed	3% (±0.01) condensed

*p<0.03 versus respective wt.

† p<0.03 versus wt-EGF.

Expression of GFP-ER_1–534_ (deleted for the F-domain and helix 12 of the E domain) resulted in PRL-array targeting with E2 treatment, but not EGF (**Figure 2** and **Table 1**). Consistent with previous observations [19], the PRL-array remained condensed in cells treated with E2 (**Figure 2**), confirming that helix 12 is necessary for E2- induced large-scale chromatin decondensation. EGF treatment of cells expressing GFP-ER_1–554_ (deleted for the F-domain) resulted in array targeting, which was indistinguishable from wild type ER (data not shown). These data exclude the involvement of the F domain in the observed differential recruitment to the PRL-array. To summarize these mapping experiments, both the AF1 and AF2 domains (helix 12) appear necessary for ER interactions with the PRL array.

Based on these differential responses, we next assayed coregulator recruitment in each setting. E2 and EGF each induced recruitment of p160 coregulators SRC-1/SRC-3, the chromatin remodeling protein BRG1, and both CDK7 and cyclin D1 (each involved in transcriptional elongation) to the PRL-array in cells expressing wild type GFP-ER (**Figure S1**). Confirming the specificity of the immunostaining reagents, cells treated with antagonists (4HT or ICI) demonstrated undetectable association of these co-regulators. Based on our set of immunological reagents, these data indicate that EGF signaling through ER recruits a similar set of coregulators at the PRL array.

### Temporal Large-Scale Chromatin Modification

To further explore E2 and EGF stimulation of ER, we performed time-course treatments (0–24 hours) and cytomorphometric analysis of large-scale chromatin modification by high-throughput microscopy (HTM). This new quantitative imaging approach [21] is a powerful tool that allows us to examine only those cells expressing a physiological level of GFP-ER (See Materials and Methods).

**Figure 3:**
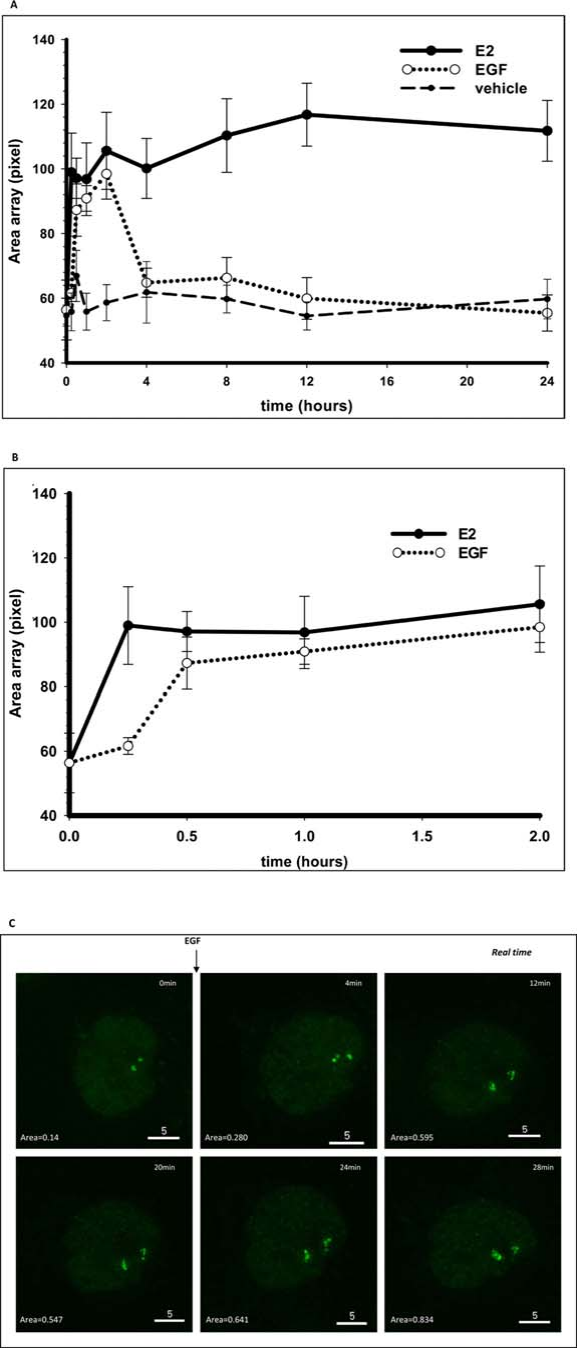
Large-scale chromatin decondensation of the PRL-array. A. PRL-HeLa cells expressing GFP-ER were treated for different times with ethanol (vehicle), E2 or EGF. After fixing and counterstaining with DAPI, cells were imaged and the array size was quantified using high throughput microscopy. B. This panel represents earlier time points to show the slower effect of EGF in inducing maximal decondensation of the array. Data represent the mean ±SEM of three different experiments. B. PRL-HeLa cells transiently expressing GFP-ER were imaged live at 37°C under constant perfusion of fresh medium (plus 100 ng/ml EGF after time 0). Image stacks were recorded every four minutes and are presented as projections. The value indicates the area of the array (µm^2^) shown in the image. Addition of EGF caused chromatin decondensation at the array within 30 minutes.

In concert with previous live cell results [19], E2 treatment is associated with a rapid decondensation of the PRL-array that reaches its maximum expansion in ∼15 minutes and, as shown in (**Figure 3A**), plateaus for at least 24 hours. In contrast, treatment of GFP-ER expressing cells with EGF resulted in a transient decondensation of the array, with full recondensation in about 2 hours (**Figure 3A**). A more detailed analysis of the early time period during expansion and contraction of the array is shown in **Figure 3B**. Note that EGF induced large-scale chromatin modification was slower than E2; ∼30 minutes for peak expansion with EGF compared to ∼15 minutes for E2. This EGF result is illustrated by time-lapse imaging of live cells shown in **Figure 3C**.

We next determined if these stimuli-induced changes in large-scale chromatin persist upon removal of E2 or EGF. Results showed that the area of the PRL-array gradually decreased, and reached basal (no treatment) condensation by approximately 2 hours after removal of E2 (**Figure S2**). Upon removal of EGF, the array experiences a steeper rate of condensation compared to E2 (**Figure S2**). Note that both E2 and EGF failed to change the size of the array in non-transfected cells, in which the array was immunolabeled with antibodies specific for RNA polymerase II or dimethlylated histone H3 (K4) (data not shown).

### ER-mediated transcriptional activity at the single cell level

**Figure 4:**
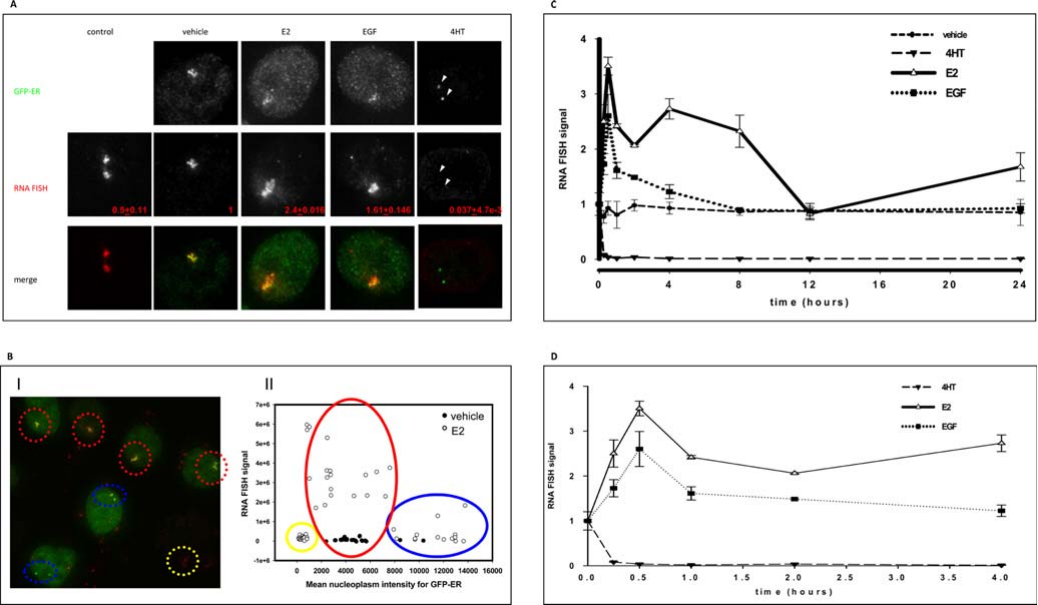
ER transcriptional activity at the promoter array. PRL-HeLa cells transiently expressing GFP-ER were treated with E2, EGF, 4-hydroxytamoxifen (4HT) or ethanolic vehicle for the indicated time. Subsequent to ligand treatment the cells were fixed and subjected to an RNA FISH protocol using a biotinylated dsRED2 probe followed by fluorescent-tagged streptavidin. A. Representative images of a single cell for each treatment. The presence of transcripts at the promoter array is identified by accumulated signal above the level for the nucleoplasm. The inset values (red type) represent the amount of transcript at 2 hours, relative to vehicle controls. B. Representative image of PRL-HeLa cells transfected with GFP-ER (green) exemplify the heterogeneity of ER expression levels. The cells show the RNA FISH signal associated with the array in the cell population (red signal) (I). The nuclear GFP-ER mean of fluorescence and RNA FISH array signal were plotted for vehicle- and E2-treated cells. Each symbol represents the measurements from a singe cell (II). C. To quantify FISH signal over 24 hours the total intensity of signal at the array (minus background signal) was determined by cumulative summation of 20 planes. Data represent the mean ±SEM of three different experiments graphed as fold induction over mean time-matched vehicle-treated control cells. D. This panel represents the earlier time points for E2 and EGF treatment. Fold activation between EGF and E2 at 30 minutes was significantly different, with a p value of 0.03.

To examine the relationship between array size and transcriptional responses to EGF, E2 or 4HT, we next used RNA FISH to measure dsRED2skl mRNA accumulation at the array (**Figure 4A**). Restorative deconvolved image stacks from GFP-ER and hybridization signals (array-associated transcripts) were collected and quantified as a measure of co-localizing reporter mRNA. First we examined the entire range of the expression within the transiently transfected cell population to determine if there was a cell-to-cell correlation between GFP-ER expression levels and the quantified FISH signal. In the PRL-HeLa cells that were non-transfected (fluorescence mean intensity was <∼500, **Figure 4B**, yellow circles), there was a low level of FISH signal detectable at the array **(** **Figure 4A** **, inset value**). Cell gating based upon ER expression level indicated a suppression of reporter transcript accumulation in cells overexpressing the receptor (GFP-ER mean intensity in the nucleus ∼>8000, **Figure 4B** **, blue circles**; intensity value >1.5fold compared to MCF-7 cells). In the remaining cells with low levels of GFP-ER (∼4000±2000, **Figure 4B** **, red circles**), FISH signals were not linearly dependent on fluorescence (receptor) levels, suggesting possible involvement of both expression level and an asynchronous cell population [22]. These observations led us to analyze cells with low levels of fluorescence (mean intensity<∼6000) in the following experiments.

In the E2 and EGF-treated cells, the mRNA transcripts at the PRL-array dramatically increased in comparison with both of the non-transfected cells and the vehicle treated cells (**Figure 4A** see the corresponding inset values). Treatment with 4HT repressed the accumulation of reporter transcripts at the PRL-array (see the arrows in the GFP-ER panel and the corresponding RNA FISH panel, **Figure 4A**) to levels below that of non-transfected cells or vehicle controls. In all cases, the transcriptional activity at the PRL-array correlated with the detection (or absence for 4HT-treated cells) of endogenous RNA polymerase II, which co-localized with the array (**Figure S3**).

We then ask: do the timing differences in the PRL-array condensation/decondensation upon E2 or EGF treatment (See **Figure 3A**) correlate with changes in transcript accumulation at the array and, more precisely, is peak chromatin decondensation at the PRL-array co-incident with maximum co-localizing transcripts? To address this question, we performed quantitative RNA FISH at regular time intervals (0–24 hours) after treatment of PRL-HeLa cells expressing GFP-ER with E2 or EGF. Results showed that EGF treatment leads to a strong, single pulse of transcript accumulation at the array ((2.8 fold of vehicle) **Figure 4C**), consistent with its ability to induce an increase in the area of the PRL-array (**Figure 3A**), both of which demonstrate similar temporal dynamics. Maximal FISH signal peaked at 30 minutes of treatment, declined sharply (approximately 40%) at one hour post treatment, and from there they gradually returned to basal (vehicle levels) by 4 hours (p>0.05, **Figure 4C**). By measuring the decay rate (λ) of the graph from an exponential curve fit, we observed that the change in array size was slower when compared with the reduction of transcriptional activity (λ = 0.026/h and λ = 0.063/h respectively).

In E2-treated PRL-HeLa cells expressing GFP-ER, a temporal analysis of RNA FISH showed a dramatically different pattern of reporter mRNA accumulation at the array (**Figure 4C**). While the PRL-array remained maximally decondensed (**Figure 3A**), the levels of reporter gene mRNA showed cyclical fluctuations over a 24-hour period (**Figure 4C**). Similar to the EGF treatment, FISH signals at the PRL-array peaked at 30 minutes post E2 treatment reaching 3.5 fold over basal levels, which is significantly higher (p = 0.03) than that induced by EGF (2.8 fold). From this point on in the 24-hour treatment period, the E2 transcription response at the PRL array was very different. The decline in FISH signal showed a slower rate of decrease compared to EGF reaching basal levels in ∼2–3 hours. At arrays that are fully decondensed (**Figure 3A**), E2 treatment induced a second peak of mRNA accumulation around 4 hours post-treatment (2.5 fold higher than non-treated control cells). By 2–3 hours after this point, transcript levels again decreased to vehicle-treated values. Interestingly, 24 hours after beginning E2 treatment, mRNA levels at the array again increased to a level ∼50% higher than control cells. Antagonist treatment (4HT) rapidly (within 15 minutes) repressed transcriptional activity, with no fluctuations (**Figure 4C**) concomitant with tight array condensation [19]. Collectively, these results identify an agonist-ligand-specific wave-pattern of mRNA accumulation at the PRL array by sustained E2 treatment versus a single pulse pattern of mRNA accumulation during EGF activation.

### Cell Signaling and ER-mediated transcription

**Figure 5:**
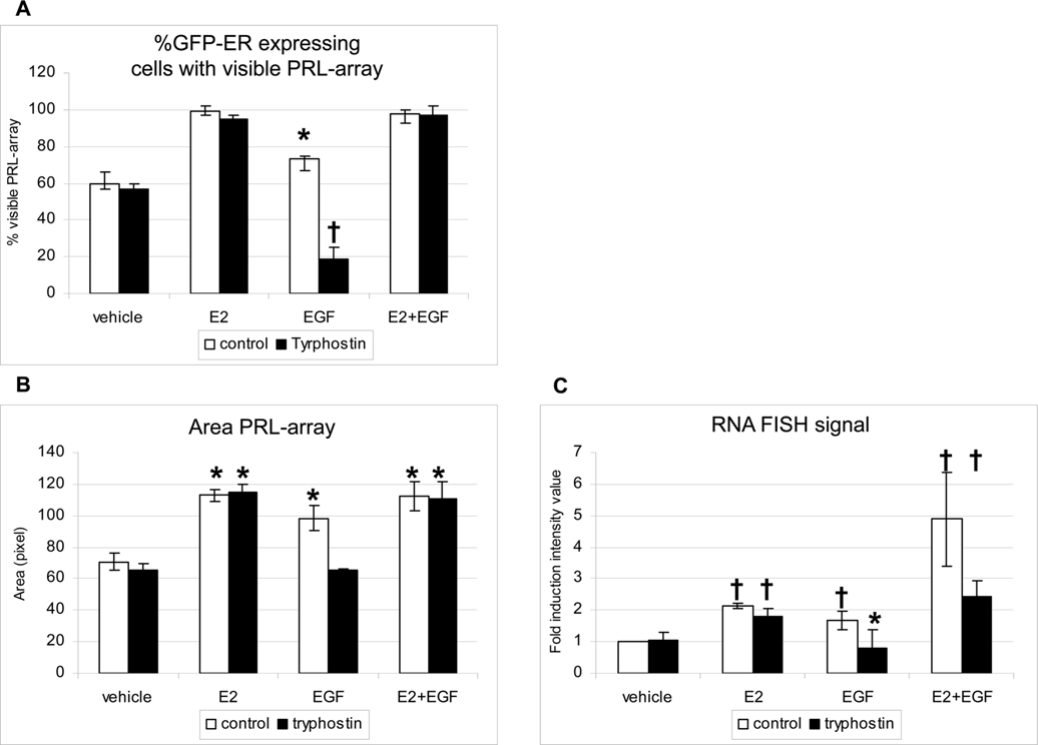
EGFR signaling and PRL array responses. PRL-HeLa cells transiently transfected with GFP-ER were pretreated or not with Tryphostin AG537 for 30 minutes before adding ethanol (vehicle), E2, EGF or E2 and EGF together for 2 hours. A. Promoter targeting: after fixing and counterstaining with DAPI, cells were imaged and the percentage of each cell population that showed visible accumulation at the array in response to treatment was calculated. Student t-test was performed for EGF treatment compared to each other treatments groups (* p<0.05, † p<0.03). B. Large-scale chromatin modification: the same images were acquired using high throughput microscopy and the array size was quantified as described in the Methods. Differences from respective vehicle treatment reaching statistical significance (p<0.05) are labeled by asterisks (*). C. Transcriptional activity: subsequent to ligand treatment, the cells were fixed and subjected to RNA FISH. The FISH signals at the array were quantified in >20 cells for each protein and treatment condition and graphed as the average total array-associated fluorescence ±SEM. Student t-test was performed for each treatment group compared to its control (* p<0.05), or to vehicle († p<0.05).

To further test EGF-dependent pathways in ER-mediated activation of the PRL array, we used an inhibitor of EGFR autophosphorylation (Tyrphostin AG537) in our assays (**Figure 5A–C**) described above. Among all cells expressing GFP-ER, EGF plus AG 537 treatments resulted in a 66% reduction (relative to EGF alone control) in array targeting (**Figure 5A**). Furthermore, array size and mRNA accumulation among the few (20%), positive cells) was similar to the control vehicle-treated cells (**Figure 5B and C**), indicating that both large-scale chromatin decondensation and reporter gene induction by ER were inhibited. As expected, the inhibitor had no effect on the GFP-ER promoter targeting by E2 (**Figure 5A**), or chromatin decondensation (**Figure 5B**) and increased transcript accumulation (**Figure 5C**).

EGF used in combination with E2 induced an increase in array size similar to that obtained with each factor separately (**Figure 5B**). Importantly, however, there was an additive effect on transcript accumulation at the array (p>0.05, **Figure 5C**), which agrees with several previous studies of reporter gene activation [3], [12], [23]. However, when PRL-HeLa cells were treated with AG537 prior to addition of E2 and EGF, transcript levels were comparable to those obtained with E2 treatment only (p>0.05, **Figure 5C**).

**Figure 6:**
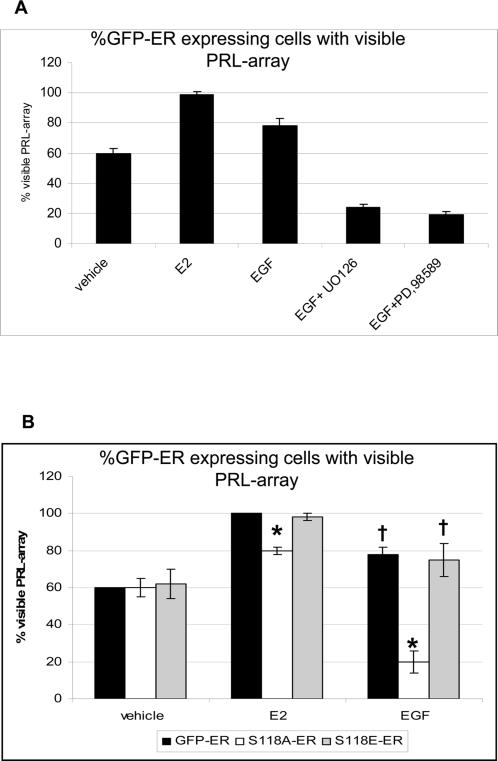
MAPK signaling and the role ER-S118 in PRL array responses. A. PRL-HeLa cells transiently expressing GFP-ER were treated with 50 µM PD98589 or 10 µM UO126 for 30 minutes before adding 10 nM E2 or 100 ng/ml EGF for 2 h. The percentage of each cell population that showed visible accumulation at the array in response to treatment was calculated and reported in the graphs (n>200). Both inhibitors dramatically reduced the number of GFP-ER-targeted arrays in transfected cells (EGF treated only) p<0.05. B. Percentage of cells with a visible array in PRL-HeLa cells transiently transfected with GFP-ER or with the two mutants, GFP-ERS118A and GFP-ERS118E, and treated with ethanol, E2 or EGF for 2 hours. Student t-test was performed for each bar compared to GFP-ER (* p<0.05), or to vehicle († p<0.05).

Since the MAPK cascade is implicated in ER transcription regulation [15], [16], we treated PRL-HeLa cells with E2 or EGF in the presence or absence of two different inhibitors of the MAPK pathway; PD98059 and UO126. Among all cells expressing a low level of GFP-ER, both inhibitors markedly decreased the percentage of cells with labeled arrays (66% reduction; **Figure 6A**). Conversely, inhibiting the MAPK pathway did not alter E2-dependent recruitment of ER to the PRL promoter array (data not shown), clearly showing that E2 and EGF operate through distinct molecular mechanisms to activate the transcriptional activity of ER.

### Serine 118 in ER-mediated PRL array responses

Serine 118 in the ER is a target residue of EGFR initiated signal transduction cascades including the MAPK pathway [14]. Conversion of this residue to a non-phosphorylable alanine (GFP-ER-S118A) strongly reduced the number of cells with ER-positive PRL-arrays after EGF treatment (75% reduction, **Figure 6B**). However, this mutation had only a minor effect on E2-dependent PRL-array targeting (20% reduction) (**Figure 6B**). Conversely, replacement by glutamic acid (S118E), which mimics phosphorylation, targeted the PRL-array similarly to wild-type ER under both conditions (E2 or EGF). Strikingly, control cells expressing GPF-ER-S118E showed no increase in the percentage of visible PRL-arrays, indicating that additional ER modification(s) may be important.

**Figure 7:**
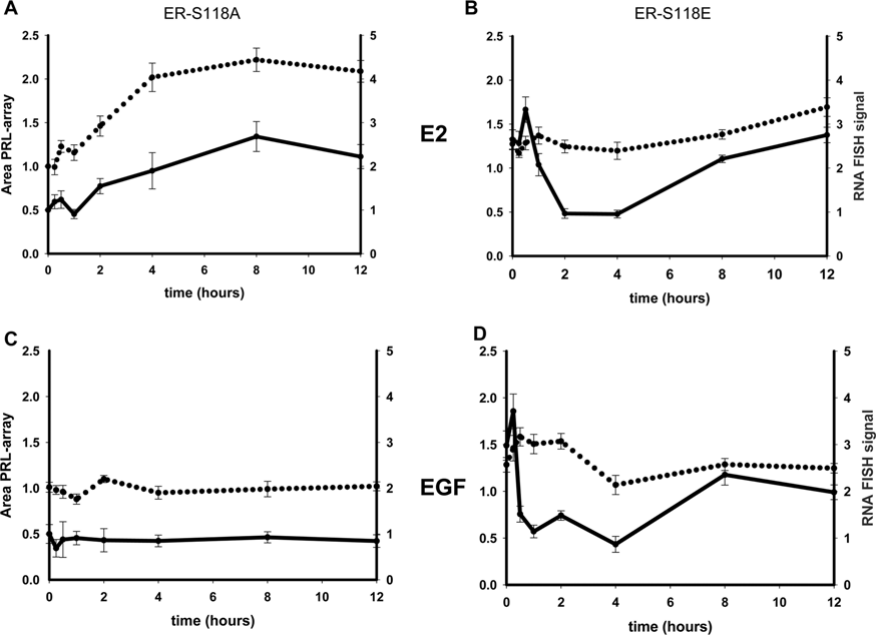
Temporal single-cell analyses of ER-S118A and ER-S118E transcriptional response at the PRL-array. PRL-HeLa cells transiently expressing the two phosphomutants, GFP-ERS118A (panel A and C) or GFP-ERS118E (panel B and D), were treated with either ethanol, E2 (panel A and B) or EGF (panel C and D) for different times. Subsequent to ligand treatment, the cells were fixed and subjected to RNA FISH using a biotinylated dsRED2 probe followed by fluorescent-tagged streptavidin. The FISH signal at the array was determined as described and the value graphed as fold induction over vehicle control cells (solid line). For each cell the area of the array was also determined and plotted as fold induction over vehicle control (dotted line). Data represent the mean ±SEM of three different experiments graphed as fold induction over time-matched vehicle-treated control cells.

We next analyzed E2- or EGF-induced large-scale chromatin modification and reporter mRNA transcription simultaneously in cells expressing GFP-ER phosphomutants. In cells transfected with GFP-ER-S118A, an increase in the level of mRNA was detected, which plateaued after 8 hours of E2 treatment, while the PRL-array was maximally decondensed after 4 hours (**Figure 7A**). In contrast, the EGF response was completely abolished in the presence of GFP-ER-S118A (**Figure 7C**).

When cells were transfected with a GFP-ER-S118E phosphomimic mutant, we observed a 1.5 fold increase in basal PRL-array size and a significantly higher basal transcript level (2.8 fold) in the absence of any stimuli (**Figure 7B and D**, **time 0**). Moreover, the responses of this mutant to E2 and EGF were very similar in terms of chromatin decondensation and transcriptional output (**Figure 7** **compare Panel B and D**). The highly decondensed status of the chromatin before treatment was also sustained for over 24 hours. Somewhat similar to the wild type ER (**see** **Figure 4C**), levels of mRNA accumulation at PRL-arrays occupied by GFP-ER-S118E exhibit two pulses of FISH signal accumulation with major peaks at 30 minutes and 12 hours for estradiol, and 15 minutes and 8 hours for EGF (**Figure 7B and D**). The presence of the phosphomutant GFP-ERS118E induces a maximum peak of transcript levels for EGF at 15 minutes (p>0.05) (**Figure 7B**). The accumulation of mRNA at 8 hours after EGF exposure was similar to E2 at the same time point (p = 0.03) but was significantly lower compared to the second peaks induced by E2 with GFP-ER wt and GFP-ERS118E (p>0.05) (**Figure 7** **)**.

## Discussion

It has been well demonstrated that EGF signaling pathways modulates ER activity [3], [14], although the molecular mechanism(s) is not clearly established. The technical complexity of cell-based assays for promoter binding, chromatin structure, and/or transcriptional activity has previously limited the focus of these investigations to each of these individually. The recent development of PRL-HeLa provides a useful tool that facilitates the study of multiple aspects of ER function at the single cell level. This includes spatiotemporal visualization of ER promoter targeting, reporter mRNA accumulation and coregulator association, in either a live or fixed cells [19]. Here, we used the PRL-HeLa model as a tool to discriminate differences in ligand-dependent and –independent regulation of ER including receptor binding to a promoter/enhancer, large-scale chromatin modification, and induction of transcription. Further, using automated image acquisition and analyses, we have specifically examined cells that expressed low levels of transfected protein, avoiding problems inherent to overexpression and bulk averaging of large populations of cells. With these approaches we report a time-dependent signature of large-scale chromatin modification and mRNA accumulation, which suggests an uncoupling of chromatin decondensation and maximal transcription.

### ER targeting at the site of transcription

The ability of a transcription factor to recognize and bind to specific elements in a promoter/enhancer is an essential step in transcription regulation. Initially, we determined that PRL-HeLa cells expressing only low levels of GFP-ER were heterogeneous in terms of unliganded ER promoter binding (60%). The responsiveness to EGF by ER in PRL-HeLa is less than E2 responses. Similar to that of E2-induced PRL-array targeting, EGF treatment resulted in a marked, but relatively sub-maximal increase in the number of cells with visible arrays (**Figure 1**). Indeed, compared to E2, EGF treatments resulted in fewer targeted PRL-arrays in GFP-ER-expressing cells. As shown in **Figure 4C**, EGF can robustly induce a PRL-reporter gene (up to ∼80% of mRNA production compared to E2 levels) in the cells showing a visible array (78%), but analyses of FISH signals in transfected cells without a visible array show a transcription level similar to non-transfected cells (data not shown and [19]). Additionally, when we examined a narrow range of GFP-ER low-expressing cells for reporter gene activity by FISH, we still observed variability in gene expression (**Figure 4B**). This supports the notion that transcriptional heterogeneity may be a stochastic process [24], [25], although transcription activation tended to peak at 30 minutes after induction (see below).

### Large-Scale Chromatin Modification and Transcription

The fundamental importance of EGF-dependent activation of ER is further supported by its ability to induce both large-scale chromatin modification and transcriptional activity over time. Relative to E2-induced activation, unliganded ER targeting the PRL-array correlates with a stable, but sub-maximal levels of chromatin decondensation and mRNA accumulation (**Figure 3A** **and** **4C**, and [19]). Our temporal studies indicate that EGF induces large-scale chromatin modification to a similar degree as E2, although at about one half the rate (30 minutes vs. 15 minutes). This result is similar to spatiotemporal changes in GFP-ER nuclear distribution observed in MCF-7 cells (10 minutes for E2 vs. 30 minutes for EGF) [26]. The physiological significance of the nuclear reorganization of GFP-ER is unknown as the concentrated foci are not frequently localized to sites of transcription [27], and have high exchange dynamics as determined by photobleaching studies [28]. Although these EGF-induced nuclear responses are alike, our PRL-array results here are directly linked to a defined transcription site. Interestingly, despite the delayed induction of large-scale chromatin modification by EGF-activated GFP-ER as compared to that by E2, the maximal level of decondensation is similar. It will be important to determine if a similar set of chromatin remodeling proteins is involved in each case, and if the recruitment of these proteins is dependent on coactivator specific post-translational modifications of ER, or specific modifications to the recruited coregulators. It will also be important to determine if these large-scale effects translate to smaller scales of chromatin structure.

After the initial decondensation, large-scale chromatin modification (as assayed by array size) induced by E2 is sustained for at least 24 hours. In contrast, continuous EGF treatment resulted in PRL-array recondensation to basal levels within a few hours. This differential regulation may be particularly important for endocrine tissues that require rapid and short responses to growth factors, in addition to the strong and sustained E2-dependent transcriptional response. The results of several studies measuring the time-course of EGFR internalization following continuous EGF treatment correlate with our observations of the temporal response of PRL-array decondensation to EGF. This suggests that the effect of EGF is modulated by down-regulation of EGFR at the plasma membrane [29], [30]. However, EGF withdrawal results in a faster rate of condensation of the PRL-array (**Figure S2**), suggesting an active yet time-limited role of the EGF-EGFR interaction in maintaining the promoter in an “open state” for transcription.

### Temporal cycling

Our previous report focused upon the early events in PRL-array transcription activity (≤2 hrs) and [19]. To obtain a more comprehensive temporal picture of ER function in response to ligand-dependent and –independent activation, we determined the relationship between large-scale chromatin alterations and mRNA accumulation over 24 hours. Our results suggest a time-dependent, stimulus-specific regulation of mRNA synthesis mediated by ER. Time course experiments show a cyclical pattern of reporter gene mRNA accumulation at the PRL-array in the continual presence of E2, which was markedly different to the non-cyclic pattern of transcription induced by sustained presence of EGF. An interesting question that we are addressing is: does ER phosphorylation cycle in phase with transcript accumulation? However, our data are in accord with data showing that P-Ser118 increased between 15 and 60 minutes following E2 treatment, as opposed to 15 and 30 minutes for EGF [31].

Cyclical ER function has been previously reported. Several groups have shown via chromatin immunoprecipitation (ChIP) analysis that levels of ER, coregulators, and RNA polymerase II crosslinked at endogenous promoters (c-Myc, pS2), can cycle at 20–40 minute intervals, dependent upon E2 treatment subsequent to transcription synchronization by promoter clearance [32]–[34]. To complement the ChIP studies, nuclear run-on assays performed on target genes [34] also revealed a significant cyclic pulse of E2-induced transcription at 45 minutes and 2 hours. Thus, both single cell studies and pooled biochemical studies demonstrate the cyclical nature of ER induction of transcription, although differing in frequency.

An intriguing *in vivo* corollary is the finding that a single injection of estradiol in ovariectomized rats at noon on day zero induced a peak of prolactin mRNA content at 8:00 PM for three days after treatment [35]. This response to estrogen is vital for a daily surge of prolactin release by the pituitary, which is important for maintenance of the corpora lutea in pregnancy [36]. Accordingly, it will be important to determine if other E2 responsive promoters such as myc and pS2 demonstrate similar cyclic activity.

The results obtained from biochemical approaches (ChIP) are obtained from millions of cells and, thus, are averaged responses that are sometimes in contrast to single cell studies, which show nuclear receptors and other transcription factors rapidly exchange with a promoter array at a much faster rate (seconds [19], [25], [37]–[40]). A reconciling model posits that transcription initiates by virtue of infrequent yet stable and productive association of factors at target genes (ChIP). These events are the result of many rapid and stochastic unproductive associations of transcription factors (FRAP). This model received substantial supportive evidence by a study of a naturally repeated gene in yeast [25]. Recent quantitative measurement in prokaryotic and eukaryotic cells also suggests that gene expression might involve intermittent pulses [41] that are stochastically regulated. Therefore, in order to achieve regulated transcription, activation-induced events must be synchronized in a spatiotemporal context. The result of synchrony would yield a higher mean transcript amount at the promoter level. Conversely, the loss of synchrony would result in periodic reduction (at 2 hrs and 12 hrs) of the mean transcription level of the population (cycling). In the context of the “visual-ChIP” system presented here, we demonstrate that synchronization is a natural process that is achieved by the addition of E2 or EGF. Furthermore, the mechanism of transcription synchronization in this model system is independent of large-scale chromatin modification.

### A disconnection between chromatin decondensation and transcription

The discoveries that chromatin structure influences transcription have expanded upon the static model based on response elements as a straightforward platform for intermediate complexes and basal transcriptional machinery [42]. The understanding of how chromatin remodeling can mechanistically affect gene regulation requires additional insight into the kinetics of the process. This includes how quickly such changes can be generated, how long they are maintained, and if they correlate precisely with transcription. Transcriptional activity, visualized here as accumulation of transcripts at the PRL-array, correlates with the presence of active transcriptional machinery on the promoter that could sterically impede DNA rewrapping before the transcriptional activity ends. The condensed status of the array chromatin visualized in 4HT treated cells (see **Figure 3A**) link both the loss of RNA polymerase II recruitment and transcription with a repressed locus while E2 and EGF induce chromatin decondensation and increase transcription at the locus. By themselves, these results suggest a direct correlation between transcription and chromatin status. However, temporal analysis of chromatin condensation and transcription indicates a more complex picture. At basal chromatin condensation (unliganded ER, EGF-treated wt-ER after 12 hrs, or ER-S118A) reporter gene mRNA accumulation is indistinguishable. Despite the constant presence of a presumably open and transcriptionally permissive state of the chromatin (wt-ER upon E2 treatment, or ER-S118E transfected cells treated with E2 and EGF), a maximal sustained level of mRNA accumulation is not observed indicating that decondensed chromatin is necessary, but not sufficient for sustained high rates of transcription. In this scenario, the presence of an open array would allow continuous sampling of the stimuli exposure. The unexpected result of Ser118E transcriptional cycling in the presence of EGF leads us to attribute the higher response of this mutant to a lack of negative feedback on its activity rather than to a stronger activation of the mutant itself.

Serine 118 of the ER plays a particularly important role both for E2 and EGF responses [16], [30]. For ligand-independent ER activation, control of phosphorylation at this site may provide an important negative feedback function on transcriptional activity and consequently on the chromatin structure at the promoter level in ER ligand-independent activation. Note in **Figure 6B**, there is a sharp reduction in targeted arrays in cells expressing GFP-ER-S118A, which have been treated with EGF. This is consistent with results from cells expressing GFP-ER that have been treated with EGF and tyrphostin (**Figure 5A**), which is discussed further below. Indeed, the phosphorylation of ER Serine 118 appears necessary to permit EGF induced large-scale chromatin modification in PRL-HeLa (**Figure 7**) and it also plays a partial role in E2-dependent activation of transcription. Results using the mutant ER-S118E receptor provide strong evidence for the critical role of phosphorylation of this residue in contributing to PRL-array modification. This mutation mimics a phosphorylated state of serine and induces a sustained decondensed PRL-array. These results suggest that ER phosphorylation alters receptor function (promoter targeting, chromatin remodeling and transcriptional output) effecting changes that could lead to a more sensitive response to EGF stimuli, as reported for tamoxifen-resistant breast tumors [43], [44]. Our results also clearly show that this residue is not only important for ER binding to the promoter, it also has a crucial role in ensuring a transcriptional response to EGF, and in regulating the temporal cycling of transcription accumulation in response to E2.

### The role of EGF signaling pathway on ER function

It is widely accepted that EGF indirectly activates ER transcription in the absence of its cognate ligand through binding to the cell membrane EGF receptor (EGFR), dimerization of the receptor and signal transduction through kinase pathways (including Ras/Raf/MAPK pathway [45]). The direct inhibition of EGFR by Tyrphostin AG537, and its downstream signal transducer, MKK1 kinase, by UO126 or PD98589 reduces EGF activation of ER, as determined by the proportion of responsive cells in our assays. Interestingly, after EGF treatment, the reduction of PRL-array targeted cells in the presence of these inhibitors was less than in control cells and is consistent with reduced array occupancy by ER-S118A (**Figure 6B**) or the AF-1 deletion mutant (**Figure 2**). This antagonist-like effect of EGF (20% versus 60%, **Table 1** and **Figure 6**) indicates the possibility that array occupancy is modulated when ER phosphorylation is indirectly, or directly, inhibited (**Figure 5** and **Figure 6** respectively). To dynamically control output signals, the EGFR signaling cascade may have evolved positive- and negative- feedback circuits [11]. The latter mechanism may participate in signal attenuation where cells can tune their response to various mixed stimuli. It is possible that when EGF and kinase inhibitor (or phosphomutant) are both present, the negative-feedback mode would dominate, perhaps by altering the phosphocode (and interactions) of ER and coregulators [46]. In this case, the end result would be a decrease in promoter targeting. In support of this argument, EGF stimulation of SRC-3 has been shown to alter its nuclear accumulation and occupancy of the PRL-array through changes in specific SRC-3 phosphorylation sites [47].

### Perspectives

The importance of transcription in cell growth and differentiation is underlined by the redundancy of feedback mechanisms that regulate those processes (different combination of events to achieve the same result in controlling gene expression). The results of this study further an emerging systems biology level view, which suggests an increased coherent understanding of gene transcription requires not only knowledge of which molecular players are involved but how they interact within the context of cell temporal/structure/organization [44], [48]. As the multiplex systems level approach described here increases in terms of automation and speed, the resultant avalanche of terabytes of image data per experiment will provide unprecedented data-mining opportunities, and challenges. Current studies focusing upon a spatiotemporal fingerprint of receptor and coregulator levels at the PRL locus, or other regulated gene arrays, should yield additional key insights into the orchestration of the molecular and cellular events involved in gene expression.

## Materials and Methods

### Cell culture

For all experiments using PRL-HeLa, cells were grown a minimum of two days in hormone-free medium containing stripped and dialyzed fetal bovine serum. Transient transfection of ER and ER mutant expression vectors was performed using the Bio-Rad Transfectin reagent following the manufacturer's protocol. After transfection the medium was replaced with ligand containing media and treated for the desired length of time.

### Immunolabeling

Antibody labeling was performed as described previously [19], [49] using 4% formaldehyde fixation (30 min) and indirect labeling with Alexa 488, 546 or 633(Molecular Probes) conjugated secondary antibodies. DAPI (1 µg/ml) was used to label DNA prior to mounting in Slow Fade (Molecular Probes). Affinity-purified rabbit antibodies to SRC-1 and SRC-3 were a kind gift from Dr. Jeimin Wong; CDK9, CyclinT1, BRG-1 and RNA Pol II large subunit were obtained from Abcam or Upstate Biotechnology and all used at 1 µg/ml for 1 hr at room temperature or overnight at 4°C.

### RNA FISH

The methods used here, including procedures for non-isotopic probe preparation and fluorescent in situ hybridization, have been published in detail [50] Briefly, coverslips with adherent cells were rinsed twice in PBS, dipped in cytoskeleton (CSK) buffer (100 mM NaCl, 300 mM sucrose, 3 mM MgCl_2_, 10 mM PIPES, pH 6.8) [51], extracted on ice for 5 minutes in CSK buffer containing 0.5% Triton X-100 and 2 mM vanadyl-ribonucleoside complex (VRC; Gibco-BRL), rinsed in CSK/VRC, fixed in 4% paraformaldehyde/PBS for 10 minutes, rinsed again in PBS and stored in 0.4% paraformaldehyde at 4°C until use. Probes substituted with biotin-labeled deoxynucleotides were made by modifications to standard nick translation procedures [50] Hybridization to RNA was carried out at 37 C in standard buffers containing 5 µg/ml probe and 50% formamide overnight. After incubation, samples were rinsed in a series of SSC buffers, assayed for biotin using streptavidin, Alexa Fluor 546 conjugate (Molecular Probes, Eugene, OR) and rinsed in a series of PBS washes. Cells were counter-stained with 1 µg/ml DAPI (Sigma, St. Louis, MO). Coverslips were then mounted on slides in Slow Fade (Molecular Probes). To quantify FISH signals, a 20 plane 128×128 (pixels), 0. 2 µm Z-stack was collected (constant exposure). Images were acquired on a Deconvolution Microscope, deconvolved and sum projected. The RNA FISH signals were quantified by using MetaMorph software (Universal Imaging, Downingtown, Pa.) after subtraction of the background nuclear fluorescence as previously described [40].Then, the integrated total RNA FISH intensity was calculated for each condition and normalized to the level of integrated total RNA FISH intensity in untreated cells to obtain the relative RNA FISH intensity. Linescans were created using SigmaPlot (Systat Software, Inc., San Jose, CA).

### Fixed Cell and Time-Lapse Imaging

The XFP-fusions and immunofluorescently labeled cells were imaged using a DeltaVision Restoration Microscopy system (Applied Precision, Issaqua, WA) and applying a constrained iterative deconvolution process. Whole nuclear volumes were collected at 0.2 µm Z steps and images from select focal planes or 3D projections were imported into Adobe PhotoShop. The Histogram adjustments were made relevant to negative controls, which routinely included non-transfected cells and/or omission of primary antibodies. Live imaging was performed by collecting short Z-stacks (∼5–10 focal planes at 300 nm increments); neutral density for the green channel was set at 50% and the images were binned 2×2. Typical exposures were for <1 sec, and time points from 3–10 minutes per stack. Projected images from each time point were used to create a QuickTime movie. Cells for live imaging were grown in 35 mm Delta T dishes (Bioptechs, Butler, PA) and secured to a stage adapter for temperature control. HEPES-buffered media was gassed overnight in a 5% CO2 incubator, and circulated through the Delta T dish using a Bioptechs peristaltic pump and inflow/outflow tubing. The temperature was controlled to 37°C (±∼0.1 degree); a Bioptechs objective heating collar was also used (also at 37°C). The Delta T dish was covered with a black plastic lid, with room for input/output tubing. For time-lapse imaging, a 63× objective (NA = 1.4) was used.

### Quantitative Image Analyses by HTM

The PRL-HeLa cells transiently expressing GFP-ER were treated with E2, 4HT, or EGF for the time indicated, fixed, and DAPI stained. The cells were imaged using the Cell Lab IC 100 Image Cytometer (Beckman Coulter) with a Nikon 40× Plan S fluor 0.90 NA objective. Two channels were imaged: channel 0 (DAPI) was used to find the focus and nuclei and channel 1 was used to image GFP-ER. A proprietary algorithm (GPCR) developed at Beckman Coulter was used to identify and quantify the GFP-ER targeted PRL-array. The parameters for the GPCR algorithm were: object scale = 30 and minimum peak height = 10. Foci identified by the GPCR algorithm are masked. The area of the mask in pixels is the measure of PRL-array size. Channel 1 was offset 2 µm from the DAPI focus for cells in all treatment conditions. This offset provided the greatest number of in focus arrays identified by the GPCR algorithm. After image acquisition and application of the GPCR algorithm the total cell populations for each treatment were progressively filtered (gated) using the same criteria. Nuclei clusters, mitotic cells, and apoptotic cells were filtered from the total cell population using an intersection of DNA content and DNA clusters gates. In addition, low GFP-ER expression and low aggregate number gates were generated and applied to produce the final cell population to be analyzed. The maximum fluorescence detected of endogenous ER in MCF-7 cell line is determined as total fluorescence in the cell. The maximum threshold for exogenous ER expression detected by immunofluorescence in PRL-HeLa cells is conservatively set to 2 times the endogenous level. The corresponding range for green fluorescence of exogenous GFP-ER is determined and used for subsequent imaging. From the final population of cells, the array size was determined using the GPCR mask. The images and masks were visually inspected for accuracy. The data was imported into SigmaPlot via an Excel Spreadsheet and fit to a single exponential decay to obtain the dissociation rate constant. Unpaired Students t-tests assuming equal variance were performed to determine statistical significance (two-tailed, p<0.05).

## Supporting Information

Figure S1 Assessment of ER ability to recruit coactivators to the promoter array. PRL-HeLa cells transiently expressing GFP-ER, were treated with 10 nM E2, 100 ng/ml EGF or ethanolic vehicle for 2 hours. Subsequent to ligand treatment the cells were fixed and endogenous coactivators (SRC-1, -3, BRG1, CDK9 and CyclinT1) detected by immunofluorescence (red). The presence of each coactivator at the promoter array is identified by accumulated signal above the level for the nucleoplasm, and representative images are illustrated here as a merge. The size bar is in microns.
(2.60 MB TIF)

Figure S2 Stimuli withdrawal regulates large-scale chromatin modification: PRL-HeLa cells were transiently transfect with GFP-ER and then treated with 10 nM E2 or 100 ng/ml EGF for 30 min (time = 0). After this time the cells were washed 5 times with PBS and the medium replaced with medium without Phenol Red and 5% SDFBS. The cells were then fixed at the indicated times, stained with DAPI and imaged with the HTM in order to quantify the array size. Results are expressed as array size normalized to control cells obtained from three independent experiments. The graph representing the cells without replacement of the stimuli (full line) is indicated for comparison. The decay rates were as follows: E2 withdrawal = 0.29/h; EGF withdrawal = 0.16/h; EGF = 0.026/h.
(0.20 MB TIF)

Figure S3 Assessment of ER ability to recruit RNA Polymerase II. PRL-HeLa cells transiently expressing GFP-ER, were treated with 10 nM E2, 100 ng/ml EGF, 10 nM 4HT or ethanolic vehicle for 2 hours. Subsequent to ligand treatment, the cells were fixed and endogenous RNA Polymerase IIo detected by immunofluorescence (red). The presence of GFP-ER and the transcription factor RNA Pol II on the promoter array is visualized as signal accumulation above the nucleoplasm level. In 4-hydroxy-tamoxifen-treated cells, RNA Polymerase IIo signal is not present at the promoter array, as identified by GFP-ER accumulation. The size bar is in microns.
(4.17 MB TIF)
