# Supplementary figures and images for: Activation of Estrogen Receptor-α by E2 or EGF Induces Temporally Distinct Patterns of Large-Scale Chromatin Modification and mRNA Transcription
Source: PLoS One. 2008 May 28;3(5):e2286. doi: 10.1371/journal.pone.0002286 (PMC2386239; doi:10.1371/journal.pone.0002286)

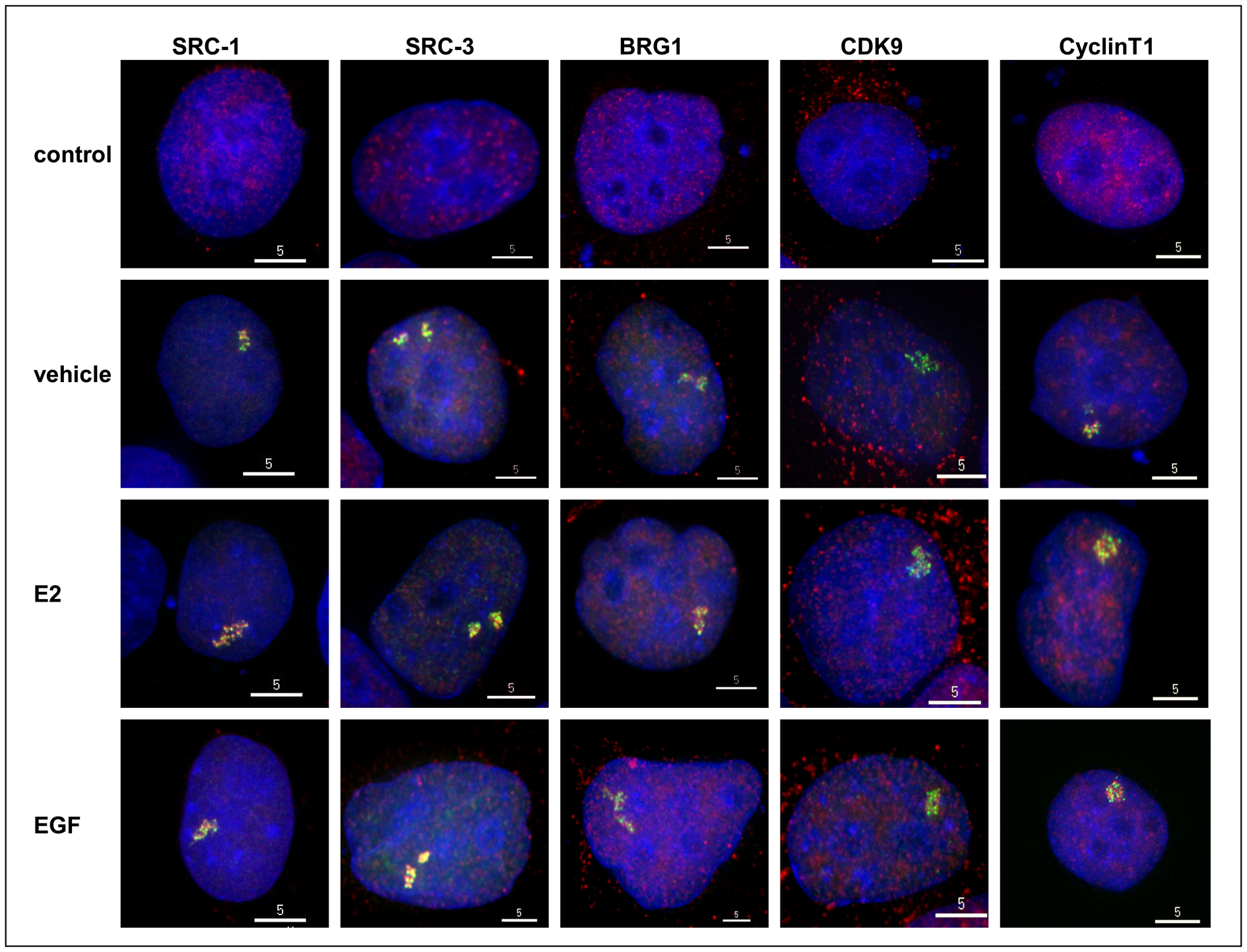

Supplement: Figure S1 — Assessment of ER ability to recruit coactivators to the promoter array. PRL-HeLa cells transiently expressing GFP-ER, were treated with 10 nM E2, 100 ng/ml EGF or ethanolic vehicle for 2 hours. Subsequent to ligand treatment the cells were fixed and endogenous coactivators (SRC-1, -3, BRG1, CDK9 and CyclinT1) detected by immunofluorescence (red). The presence of each coactivator at the promoter array is identified by accumulated signal above the level for the nucleoplasm, and representative images are illustrated here as a merge. The size bar is in microns. (2.60 MB TIF) [file pone.0002286.s001.tif]

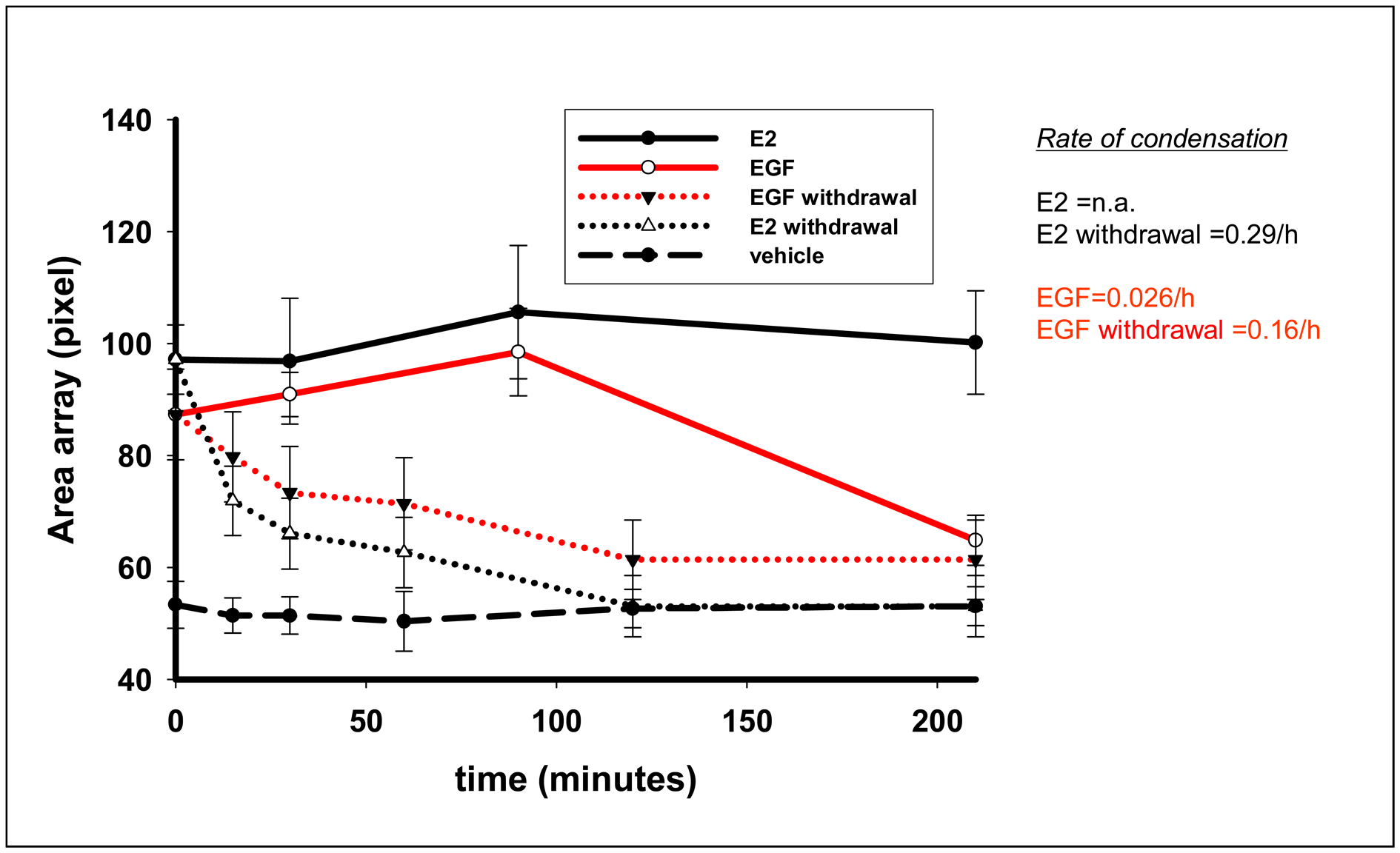

Supplement: Figure S2 — Stimuli withdrawal regulates large-scale chromatin modification: PRL-HeLa cells were transiently transfect with GFP-ER and then treated with 10 nM E2 or 100 ng/ml EGF for 30 min (time = 0). After this time the cells were washed 5 times with PBS and the medium replaced with medium without Phenol Red and 5% SDFBS. The cells were then fixed at the indicated times, stained with DAPI and imaged with the HTM in order to quantify the array size. Results are expressed as array size normalized to control cells obtained from three independent experiments. The graph representing the cells without replacement of the stimuli (full line) is indicated for comparison. The decay rates were as follows: E2 withdrawal = 0.29/h; EGF withdrawal = 0.16/h; EGF = 0.026/h. (0.20 MB TIF) [file pone.0002286.s002.tif]

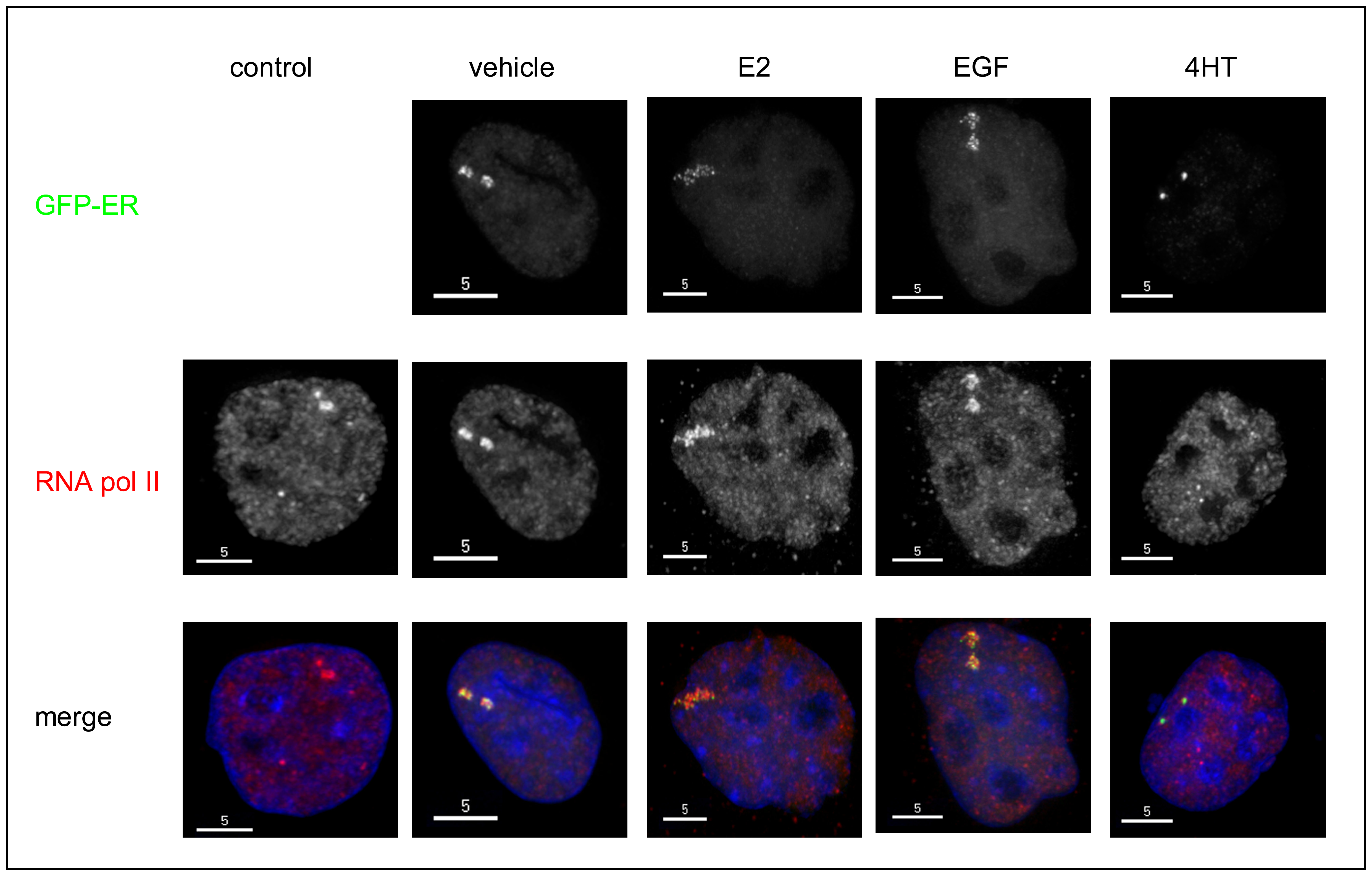

Supplement: Figure S3 — Assessment of ER ability to recruit RNA Polymerase II. PRL-HeLa cells transiently expressing GFP-ER, were treated with 10 nM E2, 100 ng/ml EGF, 10 nM 4HT or ethanolic vehicle for 2 hours. Subsequent to ligand treatment, the cells were fixed and endogenous RNA Polymerase IIo detected by immunofluorescence (red). The presence of GFP-ER and the transcription factor RNA Pol II on the promoter array is visualized as signal accumulation above the nucleoplasm level. In 4-hydroxy-tamoxifen-treated cells, RNA Polymerase IIo signal is not present at the promoter array, as identified by GFP-ER accumulation. The size bar is in microns. (4.17 MB TIF) [file pone.0002286.s003.tif]
